# Profiling Nursing Home Care Specialization Groups

**DOI:** 10.1093/geroni/igaa057.130

**Published:** 2020-12-16

**Authors:** Xiao (Joyce) Wang, Jeffrey Burr, Jennifer Hefele, Joyce Wang

**Affiliations:** 1 University of Massachusetts, Boston, Massachusetts, United States; 2 University of Massachusetts Boston, Boston, Massachusetts, United States; 3 Booz Allen Hamilton, Arlington, Virginia, United States

## Abstract

The nursing home (NH) industry has experienced a shift toward care specialization. This study used NH-level panel data from 2011 to 2017 to describe unique care specialization groups in urban areas using latent profile analysis (LPA) (N= 64695, with 12,143 unique NHs). We focused on urban NHs because NHs specialize in care due to competition and memetic pressure, more likely to be the case for urban NHs. To identify care specialization profiles, LPA was applied using different types of specialist staffing levels (physical therapist, occupational therapist, physicians, and dietitians) and the share of special care units aimed at chronic conditions like Alzheimer’s Disease and AIDs. Model diagnostics and information criterion guided selection of the best fitting model. Model stability over time, interpretability of results, and parsimony were also taken into consideration. The final results indicated a 4-profile model fit the underlying data best and the patterns remained comparatively stable over seven years. The 4-classes are uniquely identified as: high use of specialists of all types (3%), moderate use of specialists of all types (7%), mixed use of specialists and special care units (26%), and low specialization use (64%). From 2011 to 2017, the size of the ‘low specialization’ group became smaller, whereas the high and moderate groups grew larger. In addition to describing a clear trend towards increased care specialization, our findings indicated great heterogeneity in NHs’ care specialization patterns in urban areas. Future studies should examine market and organizational characteristics, as well as performance outcomes for different specialization groups.

